# Use of gene expression studies to investigate the human immunological response to malaria infection

**DOI:** 10.1186/s12936-019-3035-0

**Published:** 2019-12-13

**Authors:** Susanne H. Hodgson, Julius Muller, Helen E. Lockstone, Adrian V. S. Hill, Kevin Marsh, Simon J. Draper, Julian C. Knight

**Affiliations:** 1 The Jenner Institute, University of Oxford, Old Road Campus Road Building, Off Roosevelt Drive, Oxford, OX3 7DQ UK; 2 Department of Infectious Diseases & Microbiology, Oxford University Hospitals Trust, Oxford, UK; 3 Wellcome Centre for Human Genetics, University of Oxford, Oxford, UK; 4 Department of Tropical Medicine, University of Oxford, Oxford, UK

**Keywords:** *Plasmodium falciparum*, Gene expression, Malaria, Immunity

## Abstract

**Background:**

Transcriptional profiling of the human immune response to malaria has been used to identify diagnostic markers, understand the pathogenicity of severe disease and dissect the mechanisms of naturally acquired immunity (NAI). However, interpreting this body of work is difficult given considerable variation in study design, definition of disease, patient selection and methodology employed. This work details a comprehensive review of gene expression profiling (GEP) of the human immune response to malaria to determine how this technology has been applied to date, instances where this has advanced understanding of NAI and the extent of variability in methodology between studies to allow informed comparison of data and interpretation of results.

**Methods:**

Datasets from the gene expression omnibus (GEO) including the search terms; ‘plasmodium’ or ‘malaria’ or ‘sporozoite’ or ‘merozoite’ or ‘gametocyte’ and ‘Homo sapiens’ were identified and publications analysed. Datasets of gene expression changes in relation to malaria vaccines were excluded.

**Results:**

Twenty-three GEO datasets and 25 related publications were included in the final review. All datasets related to *Plasmodium falciparum* infection, except two that related to *Plasmodium vivax* infection. The majority of datasets included samples from individuals infected with malaria ‘naturally’ in the field (n = 13, 57%), however some related to controlled human malaria infection (CHMI) studies (n = 6, 26%), or cells stimulated with *Plasmodium* in vitro (n = 6, 26%). The majority of studies examined gene expression changes relating to the blood stage of the parasite. Significant heterogeneity between datasets was identified in terms of study design, sample type, platform used and method of analysis. Seven datasets specifically investigated transcriptional changes associated with NAI to malaria, with evidence supporting suppression of the innate pro-inflammatory response as an important mechanism for this in the majority of these studies. However, further interpretation of this body of work was limited by heterogeneity between studies and small sample sizes.

**Conclusions:**

GEP in malaria is a potentially powerful tool, but to date studies have been hypothesis generating with small sample sizes and widely varying methodology. As CHMI studies are increasingly performed in endemic settings, there will be growing opportunity to use GEP to understand detailed time-course changes in host response and understand in greater detail the mechanisms of NAI.

## Background

Malaria, caused by infection with parasites of the genus *Plasmodium*, remains a significant public health concern [1]. Despite a vaccine in pilot implementation trials [2] and widespread application of control measures [3], the disease is still responsible for a huge burden of mortality and morbidity worldwide and a concerning increase in incidence has been seen in previously well-controlled areas [3].

With repeated exposure to infection, individuals in malaria-endemic regions develop naturally acquired immunity (NAI), first to the most severe clinical forms, such as cerebral malaria and then more slowly to infection itself [1]. Although the role of antibodies in controlling parasite density, symptomatology and severity of disease is well established [4, 5], less is known about mechanism in terms of the role of the innate and cellular immune responses [6]. Increased understanding of the immune response to malaria, in particular those that mediate NAI, could aid identification of diagnostic and prognostic markers, inform vaccine development and assist with the identification of treatment strategies to modify the immunological mechanisms mediating severe pathology [1].

Transcriptomics, which allows the expression of thousands of genes to be assessed in parallel for a single RNA sample, is an exciting, expanding area of research with vast potential application in the field of infection [7]. Facilitating a systems biology approach, gene expression data from high-throughput technologies (such as microarrays [8] and next generation sequencing enabling RNA sequencing for bulk cell populations and at single-cell resolution [9, 10]) can allow greater understanding of individuals’ response to infection. To date, expression data have been used to dissect mechanisms of vaccine immunogenicity [11], inform the design of new vaccines [12, 13], predict response to infection and outcome [14, 15], characterize and improve understanding of sepsis [16], and offer a novel approach to the diagnosis of infectious pathogens [17–19] together with RNA expression in the pathogen [20].

Given the limited understanding of the mechanisms of NAI to malaria from traditional immunological studies, a systems approach characterizing the gene expression patterns associated with infection could provide novel and valuable insights [21, 22]. Transcriptional profiling of the immune response to malaria in humans to date has sought to identify markers to aid diagnosis [23], to understand the pathogenicity of severe disease [24] and dissect the mechanisms of NAI [25, 26]. However, interpreting this body of work is difficult given considerable variation in study design, definition of disease, patient selection and methodology employed.

This review outlines a comprehensive analysis of all GEP studies of the human immune response to malaria with two aims: (i) to understand the application of this technology to date, in particular how these studies have informed understanding of NAI; and (ii) to determine the extent of variability in methodology between studies to allow informed comparison of data and interpretation of results.

## Methods

A search of Gene Expression Omnibus (GEO) [27] for datasets including the search terms; ‘plasmodium’ or ‘malaria’ or ‘sporozoite’ or ‘merozoite’ or ‘gametocyte’ and ‘Homo sapiens’ was performed on 10th September 2019. Each of these datasets were examined and those not relating to the human immune response to malaria infection or using the *Homo sapiens* platform excluded. Of note, datasets of gene expression changes in relation to malaria vaccines were excluded.

## Results

### Studies identified

The search identified 30 GEO datasets. Seven of these datasets were excluded, as published analyses were unavailable. Twenty-three datasets and 25 related publications were therefore included in the final review (Table 1 and Additional file 1: Figure S1). All datasets related to *Plasmodium falciparum* infection except two that related to *Plasmodium vivax* infection (Table 1). The majority of datasets included samples from individuals infected with malaria ‘naturally’ in the field (n = 13, 57%), however some related to controlled human malaria infection (CHMI) studies (n = 6, 26%), or cells stimulated with *Plasmodium* in vitro (n = 6, 26%). Studies included samples from individuals with a wide range of ages (from 2 months—varying ages of adulthood) with differing degrees of prior exposure and, therefore, NAI to malaria. Samples were often collected as part of wider immuno-epidemiological studies or vaccine trials, leading to variation in study design and sampling intervals.

**Table 1:** Summary of gene expression datasets investigating the human immunological response to malaria infection

GEO series	Title of dataset	Publication	Design	Infection/antigenic Stimulation	Species	Tissue	Age	Participant origin	Expression profiling	Subjects (samples)^a^	Controls	Platform name	Platform technology
GSE2900	Host response malaria	Griffiths et al. (2005)	Comparison of GEP in febrile children with convalescent samples 2 weeks post discharge	Field	*P. falciparum*	Whole blood: PAX gene	Children 2–126 months	Kenya	Array	22 (28)	Subject paired samples: diagnosis and post treatment	LC-36	Spotted DNA/cDNA
GSE5418	Gene expression analysis in malaria infection	Ockenhouse et al. (2006)	Comparison of GEP in early, pre-symptomatic blood-stage infection post CHMI with symptomatic malaria-experienced adults with naturally acquired malaria	CHMI and Field	*P. falciparum*	PBMC	Adults; 19–49 years	USA and Cameroon	Array	37 (74)	22 un-infected malaria-naïve American adults	Affymetrix human genome U133A array	In situ oligonucleotide
GSE15221	Malaria primes the innate immune response due to IFNγ induced enhancement of Toll-like receptor expression and function	Franklin et al. (2009) and Sharma et al. (2011) and Hirako et al. (2018)	Comparison GEP at malaria diagnosis and 28 days post treatment	Field	*P. falciparum*	PBMC	Adults 30 ± 10 years	Brazil; Porto Velho	Array	21 (42)	Subject paired samples: diagnosis and post treatment	Illumina human-6 v2.0	Oligonucleotide beads
GSE26876	Time kinetics of gene expression in NK92 cells after *P. falciparum*-iRBC encounter	De Carvalho et al. (2011)	Comparison of GEP variation of NK92 cells after 6, 12, and 24 h of co-culture with either infected or uninfected RBC compared to time-point 0	In vitro—iRBC	*P. falciparum*	NK92 cell line	N/A	N/A	Array	N/A (12)	Paired samples: pre and post exposure	Affymetrix human gene 1.0 ST array	In situ oligonucleotide
GSE33811	Paired whole blood human transcription profiles from children with severe malaria and mild malaria	Krupka et al. (2012)	Comparison of GEP in severe malaria and subsequent mild malaria in same subjects 1 month later	Field	*P. falciparum*	Whole blood: tri-reagent BD	Children: 8–45 months	Malawi	Array	5 (10)	Subject paired samples: severe and mild malaria	Affymetrix Human Gene 1.0 ST Array	In situ oligonucleotide
GSE34404	The genomic architecture of host whole blood transcriptional response to malaria infection	Idaghdour et al. (2012)	Comparison of GEP in mild malaria with age matched un-infected controls	Field	*P. falciparum*	Whole blood: Tempus	Children; median age 3.7 years	Benin	Array	94 subjects (94) and 64 controls (64)	Uninfected age matched	Illumina HumanHT-12 V4.0 expression bead chip	Oligonucleotide beads
GSE55843	Loss and dysfunction of Vdelta2 + gamma delta-low T cells is associated with clinical tolerance to malaria	Jagannathan et al. (2014)	Comparison of GEP of Vδ2 + T cells from children with ‘high’ and ‘low’ episodes of malaria in the preceding year	In vitro—iRBC	*P. falciparum*	Vδ2 + T cells	Children: 4–5 years	Uganda	Array	78 (156)	N/A	Agilent-039494 SurePrint G3 Human GE v2 8 × 60K Microarray 03938	In situ oligonucleotide
GSE53292	Transcriptomic analysis of Plasmodium PBANKA, PBSLTRiP-KO, PB268-KO parasite infected and uninfected host cell	Jaijyan et al. (2015)	Comparison of GEP of uninfected HepG2 with those infected with wild-type and knock out sporozoites	In vitro—sporozoites	*P. falciparum*	HepG2 cells	N/A	N/A	High throughput sequencing	NK	NK	Illumina Genome Analyzer IIx (Homo sapiens)	High-throughput sequencing
GSE50957	Molecular hallmarks of experimentally acquired immunity to malaria [Pilot Study]	Tran et al. (2016) and Vallejo et al. (2018)	Comparison of GEP pre and post infection	CHMI	*P. falciparum*	Whole blood: PAX gene	Adults: 19–22 years	USA	High throughput sequencing	5 (10)	Subject paired samples: Pre and post infection	Illumina HiSeq 2000 (Homo sapiens)	High-throughput sequencing
GSE52166	Molecular hallmarks of naturally acquired immunity to malaria	Tran et al. (2016)	Comparison of GEP pre and post infection	Field	*P. falciparum*	Whole blood: Tempus	Adults and Children 13.5–23.3 years	Malawi	High throughput sequencing	8 (16)	Paired same subject pre infection	Illumina HiSeq 2000 (Homo sapiens)	High-throughput sequencing
GSE64338	Expression data from whole blood samples of Rwandan adults with mild malaria with matched sample 30 days later (convalescence)	Subramaniam et al. (2015)	Comparison of GEP in mild malaria and 30 days later	Field	*P. falciparum*	Whole blood: Tri-Reagent BD	Adults	Rwandan	Array	19 (38)	Subject paired samples: diagnosis and post treatment	[HuGene-1_0-st] Affymetrix Human Gene 1.0 ST Array	In situ oligonucleotide
GSE64493	FCRL5 delineates functionally impaired memory B cells associated with malaria exposure	Sullivan (2015)	Comparison of GEP between classical and atypical memory B cells in Uganda children	Field	*P. falciparum*	PBMC	Children 8–10 years	Uganda	Array	12	NK	Agilent-039494 SurePrint G3 Human GE v2 8 × 60K Microarray 039381	In situ oligonucleotide
GSE67184	Transcription profiling of malaria-naïve and semi-immune colombian volunteers in a *Plasmodium vivax* sporozoite challenge	Rojas-Penas (2015), Vallejo (2018) and Gardinassi (2018)	Comparison of GEP changes between malaria naïve and semi-immune adults pre-infection and at diagnosis	CHMI	*P. vivax*	Whole blood: Tempus	Adults	Columbia	High throughput sequencing	12 (24)	Subject paired samples: pre-infection and diagnosis	Illumina HiSeq 2500 (Homo sapiens)	High-throughput sequencing
GSE67469	Transcription profiling of malaria-naïve and semi-immune colombian volunteers in a *Plasmodium vivax* sporozoite challenge	Rojas-Penas (2015)	Comparison of GEP changes between malaria naïve and semi-immune adults over the time-course of malaria infection: pre-infection, day 5, day 7, day 9, diagnosis and month 4	CHMI	*P. vivax*	Whole blood: Tempus	Adults	Columbia	RT-qPCR	16 (85)	Subject paired samples: Pre infection and multiple time-points post infection	Fluidigm 96Ã—96 nanofluidic arrays for 96 genes: blood informative transcripts	RT-PCR
GSE7586	Genome wide analysis of placental malaria	Muehlenbachs (2007)	Comparison of GEP in women with placental malaria and those without	Field	*P. falciparum*	Placenta	Adults	Tanzania	Array	20 (20)	NK	[HG-U133_Plus_2] Affymetrix Human Genome U133 Plus 2.0 Array	In situ oligonucleotide
GSE77122	Involvement of β-defensin 130 (DEFB130) in the macrophage microbicidal mechanisms for killing *Plasmodium falciparum*	Terkawi (2017)	Human monocyte-derived macrophages were co-cultured with *P. falciparum* iRBCs, saponin-treated iRBCs, or non-infected RBCs	In vitro—iRBC	*P. falciparum*	Macrophages	NK	NK	Array	NK (8)	NK	Agilent-028004 SurePrint G3 Human GE 8 × 60K Microarray	In situ oligonucleotide
GSE93664	Comparison of the transcriptomic profile of *P. falciparum* reactive polyfunctional and IFNγ monofunctional human CD4 T cells	Burel (2017)	Comparison of GEP in monofunctional and polyfunctional IFN producing T cells collected 21 days post CHMI infection	CHMI + in vitro—iRBC	*P. falciparum*	IFN producing T cells	18–42 years	Australia	Array	8 (2)	NK	[HuGene-2_0-st] Affymetrix Human Gene 2.0 ST Array	In situ oligonucleotide
GSE100562	RNA-sequencing analysis of response to *P. falciparum* infection in Fulani and Mossi ethnic groups, Burkina Faso	Quin (2017)	Comparison of GEP in onocytes and CD14− cells in *P. falciparum* infected and uninfected malaria-exposed Fulani and Mossi sympatric ethnic groups	Field	*P. falciparum*	Monocytes (CD14+) and lymphocytes (CD14−)	15–24 years	Burkino Faso	High throughput sequencing	23 (23)	NK	Illumina HiSeq 2500 (Homo sapiens)	High-throughput sequencing
GSE1124	Whole blood transcriptome of childhood malaria	Boldt (2019)	Comparison of GEP of children with asymptomatic parasitemia, uncomplicated malaria, malaria with severe anaemia and cerebral malaria	Field	*P. falciparum*	Whole blood: PAX gene	0.5–6 years	Gabon	Array	NK	Healthy control children	[HG-U133A] Affymetrix Human Genome U133A Array	In situ oligonucleotide
GSE114076	Differential gene expression profile of human neutrophils cultured with *Plasmodium falciparum*-parasitized erythrocytes	Terkawi (2018)	Comparison of GEP in neutrophils incubated with iRBC or non-infected RBC	In vitro—iRBC	*P. falciparum*	Neutrophils	NK	NK	Array	1 (8)	Culture with non-infected RBC	Agilent-072363 SurePrint G3 Human GE v3 8 × 60K Microarray	In situ oligonucleotide
GSE97158	Transcriptional responses induced by controlled human malaria infection (CHMI)	Rothan (2018)	Comparison of GEP in whole blood pre and post sporozoite CHMI in malaria exposed adults	CHMI	*P. falciparum*	Whole blood: PAX gene	Adults	Tanzania	High throughput sequencing	10 (40)	Subject paired samples: pre and post CHMI	Illumina HiSeq 2000 (Homo sapiens)	High-throughput sequencing
GSE65928	Malaria-associated atypical memory B cells exhibit markedly reduced B cell receptor signaling and effector function	Portugal (2015)	Comaprison of GEP of naïve B cells, classical and atypical memory B cells in immune adults	Field	*P. falciparum*	B cells	Adults: 18–37 years	Mali	Array	20 (20)	US healthy adults	[HuGene-2_0-st] Affymetrix Human Gene 2.0 ST Array [transcript (gene) version]	In situ oligonucleotide
GSE72058	Activated neutrophils are associated with pediatric cerebral malaria vasculopathy in Malawian children	Feintuch (2016)	Comparison of GEP in cerebral malaria between children with malaria retinopathy and those without	Field	*P. falciparum*	Whole blood: Tri-Reagent BD	Children 6 month–12 years	Mali	Array	98 (98)	NK	[HuGene-1_0-st] Affymetrix Human Gene 1.0 ST Array [transcript (gene) version]	In situ oligonucleotide

*PBMC* peripheral blood mononuclear cells, *GEP* gene expression profile, *CHMI* controlled human malaria infection, *iRBCs* infected red blood cells, *N/A* not applicable, *NK* not known

^a^Samples analysed for publication

### Review of methodological approaches

**Fig. 1:**
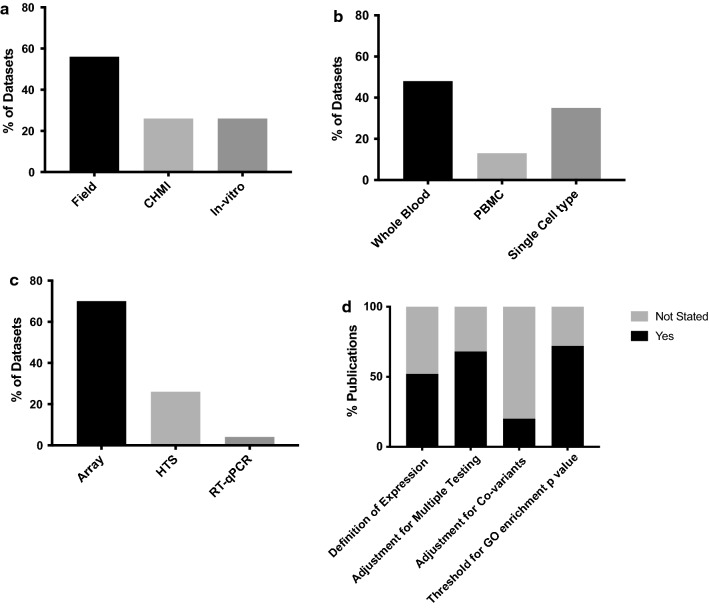
Comparison of key methodological variables between datasets or publications. **a** Antigenic stimulation; *CHMI* controlled human malaria infection, *‘field’* infection naturally by mosquito bite, *‘in-vitro’* in vitro stimulation by sporozoites or infected red blood cells. Some datasets employed more than one method of antigenic stimulation. **b** Tissue type analysed; *PBMC* peripheral blood mononuclear cells. **c** Expression profiling method: *HTS* high throughput sequencing. **d** Manipulation of data, *go* gene ontology

Significant heterogeneity in the datasets was found in terms of study design, sample type, platform used and method of analysis (Tables 1, 2 and Fig. 1), making direct comparison of results between studies difficult. Most datasets were generated from whole blood samples (n = 11, 48%), however some used PBMCs (n = 3, 13%) or individual tissue or cells types (n = 8, 35%) (Table 1). For the majority of studies, expression profiling was performed by array (n = 16, 70%), with others using high throughput sequencing (n = 6, 26%) or RT-qPCR [28] (n = 1, 4%) (Table 1). There was heterogeneity in data generation between studies with variation in methods used for normalization of data and adjustment for co-variables (Table 2). Thresholds for significance varied considerably and not all studies applied corrections for multiple testing. Choice of database used for gene ontology analysis also varied and there was variable, often incomplete reporting of analysis methods used (Table 2).

**Table 2:** Comparison of methodological approaches for analysis of gene expression data

Dataset		Data generation									Gene ontology analysis			
GEO series	Publication	RNA Quantification Platform	Normalization	Adjustment for covariates	Definition expression	Expressed genes	Threshold FC	Threshold P	Test	Multiple testing	GO analysis	Threshold GO enrichment p	Test	Multiple testing
GSE2900	Griffiths (2005)	Stanford University cDNA lymphochip two color microarray	Scaled to geometric mean of sample:reference signal ratio from all array features	NS	Signal threshold	9869	2.5 (from median in > 4 samples)	0.1	Permutation	FDR	NA	NA	NA	NA
GSE5418	Ockenhouse (2006)	Affymetrix U133A GeneChips	RMA	NS	NS	NS	No	0.01	SAM, t-test	FDR	Onto Express and Pathway Architect	*0.05*	NS	FDR
GSE15221	Franklin (2009) and Sharma (2011)	Illumina Human WG-6 v2.0	Cubic spline	NS	Signal threshold	NS	1.7	0.01	Paired t-test	FDR	Onto Express	Varying	NS	NS
GSE15221	Hirako (2018)	Illumina Human WG-6 v2.0	Cubic spline	NS	Signal threshold	NS	1.5	0.01	Permutation and t-test	FDR	DAVID, GSEA	0.05	Multiple	FDR
GSE26876	de Carvalho (2011)	Affymetrix Human Gene 1.0 ST Array	RMA	NS	NS	NS	1.5	0.05	Student t-test	No	Ingenuity pathway analysis	NS	NS	NS
GSE33811	Krupka (2012)	Affymetrix Human Gene 1.0 ST Array	RMA and Quantile	NS	Signal and variation threshold	3110	2	0.05	Paired t-test	No	Gene set enrichment analysis on selected GO terms	*0.01*	Paired t-test	FDR
GSE34404	Idaghdour (2012)	Illumina Human HT-12 BeadChips	Quantile	Location, Sex, Hb, total cell counts (RBCs and WBCs) and ancestry	Signal and normality threshold	NS	2 (for comparison)	0.01	ANOVA, ANCOVA	FDR	Gene set enrichment analysis on customized MsigDB database	*0.05*	NS	Bonferroni
GSE55843	Jagannathan (2014)	Agilent Sure Print G3 Human Gene Expression 8 × 60K v2 gene expression microarrays	Quantile	NS	Signal threshold	NS	2	0.05	SAM	FDR	NA	NA	NA	NA
GSE53292	Jaijyan (2015)	Illumina Genome Analyzer Iix 72SE	NS	NS	NS	NS	NS	0.05	t-test	No	GeneCodis3, Bingo 2.3 plugin (Cytoscape 2.8.3)	*0.05*	NS	NS
GSE50957 GSE52166	Tran (2016)	Illumina HiSeq 2000 2 × 100 PE	TAMM	Batch, Sex, Age, Pre-infection baseline	Signal and variation threshold, removal Y chromosomes	NS	1.5	0.05	Limma	FDR	Ingenuity pathway analysis	*0.05*	Fisher exact test	FDR
GSE50957 GSE67184	Vallejo (2018)	Illumina HiSeq 2000 2 × 100 PE	CPM, TPM	NS	Signal threshold	NS	NS	0.05	EdgeR	FDR	WGSEA, ToppGene, STRING	*0.05*	Multiple	FDR
GSE64338	Subramaniam (2015)	Affymetrix Human Gene 1.0 ST Array	Nonlinear normalization based on Li-Wong methods	NS	NS	NS	1.2	0.001	Paired t-test	FDR	Ingenuity Pathway Analysis	*0.05*	NS	FDR
GSE64493	Sullivan (2015)	Agilent Sure Print G3 Human Gene Expression 8 × 60K v2 gene expression microarrays	Quantile	NS	Signal threshold	NS	1.5	0.03	Limma	FDR	DAVID	*0.05*	NS	FDR
GSE67184	Rojas-Penas (2015)	Illumina HiSeq 2500 2 × 100 PE	SNM	Location/time-point, subject (random effect)	Signal threshold	6154	No	0.05	NS	FDR	NA	NA	NA	NA
GSE67184	Gardinassi (2018)	Illumina HiSeq 2500 2 × 100 PE	NS	NS	NS	NS	No	0.05	Limma, repeated measures ANOVA	FDR	GSEA on blood transcriptome modules (BTM, Li et al.)	*0.05*	permutation	FDR
GSE7586	Muehlenbachs (2007)	Affymetrix U133 Plus 2.0 GeneChip	GC RMA	NS	NS	NS	2.5	0.01	t-test	No	NA	NA	NA	NA
GSE77122	Tarawa (2017)	Agilent Sure Print G3 Human Gene Expression 8 × 60K gene expression microarrays	Each gene expression array dataset was normalized to the in silicon pool for the macrophages cultured with RBCs	NS	NS	NS	No	0.05	Paired t-test	No	DAVID	*0.05*	Fisher exact test	No
GSE93664	Burl (2017)	Affymetrix Human Gene ST 2.0 gene array	RMA	NS	NS	NS	2	0.05	NS	No	STRING	*0.01*	NS	Corrected unspecified
GSE100562	Quin (2017)	Illumina HiSeq 2500 2 × 50 PE	NS	NS	NS	NS	No	0.05	Limma	FDR	NA	NA	NA	NA
GSE1124	Boldt (2019)	Affymetrix U133A + B GeneChips	RMA	NS	Signal threshold	NS	1.9	0.004	SAM	FDR	DAVID and Ingenuity Pathway Analysis	*0.05*	NS	NS
GSE114076	Terkawi (2018)	Agilent Sure Print G3 Human Gene Expression 8 × 60K gene expression microarrays	Each gene expression array dataset was normalized to the in silicon pool for the neutrophils cultured with RBCs	NS	NS	NS	2	0.01	Limma	No	Genomatix GeneRanker, DAVID, NET-GE and Enricher	*0.05*	NS	Corrected unspecified
GSE97158	Rothan (2018)	Illumina HiSeq 2500 2 × 51 PE	TMM	Blocking by subject, in two separate models interaction with cell count and time of parasitemia was added	Signal threshold	16,473	1.5	0.05	Limma	FDR	GSEA (camera) on blood transcriptome modules (BTM, Li et al.)	*0.05*	Fisher exact test	FDR
GSE65928	Portugal (2015)	Affymetrix Human Gene ST 2.0 gene array	RMA	NS	NS	NS	NS	0.05	ANOVA	FDR	Ingenuity pathway analysis	NS	NS	NS
GSE72058	Feintuch (2016)	Affymetrix Human Gene 1.0 ST array	RMA and Quantile	Peripheral parasitemia	NS	NS	No	0.05	t-test	No	GSEA, CateGOrizer and ingenuity pathway analysis	0.2 and 0.06	NS	FDR

*FDR* false discovery rate, *Hb* haemoglobin, *NA* not available, *NS* not specified in publication, *RBCs* red blood cells, *RMA* Robust Multichip average, *SNM* supervised normalization of microarray, *TMM* trimmed mean of M-values, *GEO* Gene Expression Omnibus, *GE* gene ontology

### Transcriptional insights into the immune response to malaria infection

Seven datasets provided insight into the transcriptional changes associated with NAI to malaria (Table 3) [24–26, 28–31]. However, given the difficulty in defining or quantifying NAI for an individual, studies varied in their approach, choosing to examine GEPs in settings of varying history of prior exposure to malaria [25, 26, 28, 29], symptomatology during infection [25] or severity of disease [24, 32]. All studies examining NAI included small numbers of subjects and all deployed different experimental designs (Table 3).

**Table 3:** Gene expression studies informing understanding of naturally acquired immunity to malaria infection

Measure of NAI	Publication	Design	Sample	Species	Subjects for comparison		Key finding	Comment
Prior exposure to malaria	Tran et al. (2016)	Comparison of GEP changes from paired infected and uninfected samples	Whole blood	*P. falciparum*	Malaria-naïve, symptomatic Dutch CHMI volunteers at diagnosis (n = 5)	Malaria experienced Malian children (> 13 years) and adults infected in the field (n = 8)	Graded activation of pathways of downstream proinflammatory cytokines with highest activation in malaria-naive subjects and significantly reduced activation in malaria experienced Malians	
	Ockenhouse et al. (2006)	Comparison of GEP changes in infection-controls samples US malaria naïve subjects	PBMC	*P. falciparum*	US malaria-naïve CHMI volunteers with early, blood-stage infection (n = 22)	Malaria-experienced Cameroonian adults presenting with naturally acquired febrile malaria (n = 15)	Similar induction of pro-inflammatory cytokines seen between pre-symptomatic and symptomatic individuals regardless of prior malaria exposure	
	Rojas-Pena et al. (2015) and Vallejo et al. (2018)	Comparison of GEP changes from paired infected and uninfected samples	Whole blood	*P. vivax*	Columbian malaria-naïve (MN) CHMI volunteers at diagnosis (n = 7)	Columbian malaria-exposed (ME) CHMI volunteers at diagnosis (n = 9)	Little differentiation seen between MN and ME populations by Rojas-Penas et al. However network co-expression analysis by Vallejo et al. showed the inflammatory response was attenuated in ME volunteers with decreased class II antigen presentation in dendritic cells	No significant difference between groups for pre-patent period or parasitaemia at diagnosis suggesting there may have been no difference in functional immunity between groups
	Jagannathan et al. (2014)	Comparison of GEP between groups	Vδ2^+^ T cells	*P. falciparum*	Ugandan children with low prior malaria incidence (n = 4)	Ugandan children with low prior malaria incidence (n = 4)	Comparison of basal gene expression patterns of sorted, un-stimulated Vδ2^+^ T cells identified 48 differentially expressed genes, many with known roles in immunomodulation. For each of these genes, expression was higher among children with high prior exposure to malaria	Data suggest recurrent malaria infection causes up-regulation of immunoregulatory pathways that dampen the pro-inflammatory immune response to *P. falciparum* infection and help explain immunological tolerance to the parasite
Symptoms at diagnosis	Tran et al. (2016)	Comparison of GEP changes from paired infected and uninfected samples	Whole blood	*P. falciparum*	Malaria experienced Malian children (> 13 years) and adults infected in the field and asymptomatic at diagnosis (EA, n = 5)	Malaria experienced Malian children (> 13 years) and adults infected in the field and symptomatic with fever at the time of diagnosis (EF, n = 3)	Only 70 differentially expressed genes (DEGs) were identified between these groups despite the apparent clinical differences	2 of the 5 individuals in the EA group progressed to febrile malaria within 5 days of initial diagnosis by PCR
Disease severity	Krupka et al. (2012)	Comparison of GEP in same subjects at diagnosis with severe and subsequent mild malaria	Whole blood	*P. falciparum*	Malawian children who, after presenting with severe malaria (all had cerebral malaria), were found to have mild malaria one month later on screening by blood smear (n = 5)		Pathway analysis showed relative up regulation of Type I IFN signaling pathway, regulation of inflammation, regulation of leukocyte proliferation and T cell activation in episodes of mild malaria	
	Boldt et al. (2019)	Comparison of GEP between groups	Whole blood	*P. falciparum*	Healthy uninfected Gabonese children	Gabonese children with asymptomatic parasitaemia, mild malaria, malaria with severe anaemia and cerebral anaemia (0.5–6 years)	GEP of 22 genes significantly differed among groups. Immunoglobulin production, complement regulation and IFN beta signaling were most conspicuous	

*PBMC* peripheral blood mononuclear cells, *GEP* gene expression profile, *CHMI* controlled human malaria infection

The findings from a number of studies supported a dampening of the innate pro-inflammatory immune response as a mechanism underpinning NAI [24–26, 33] although this finding was not observed in all studies [28, 29, 31].

One study by Franklin et al. provided evidence of ‘pro-inflammatory priming’ of the innate immune system in acute malaria infection [34]. Comparison of GEP in Brazilian adults presenting with uncomplicated malaria with paired convalescent samples showed an increase in expression in genes involved in TLR signalling pathways supporting a role for TLR hyper responsiveness in the pathology of malaria infection [34, 35].

Quin et al. sought to use RNA sequencing to elucidate the mechanism driving lower infection rates, lower parasite densities and fewer symptomatic cases of *P. falciparum* in the population of Fulani compared to other sympatric ethnic groups [33]. Comparison of the GEP of monocytes from infected and uninfected Fulani and Mossi adults showed a marked difference, with a significantly greater number of differentially expressed (DE) genes in infected Fulani compared to infected Mossi participants (1239 versus 3 DE genes respectively). Pathway analysis showed that infected Fulani, but not infected Mossi, individuals demonstrated a marked reduction in expression of inflammasome pathway components, suggesting a blunting of the innate pro-inflammatory immune response post-infection could explain the differences in susceptibility.

Another study sought to examine the genetic basis of gene expression variation in malaria [36]. Idaghdour et al. compared GEP in children diagnosed with uncomplicated malaria (n = 94) in Benin with age matched controls (n = 64) [36] and performed a genome wide association test of transcript abundance. Testing for genotype-by-infection interactions demonstrated the existence of genome wide significant interactions and other genes subject to interaction effects beneath genome-wide significance but still likely to have important roles in modulating the course of infection. These interactions affected the complement system, antigen processing and presentation and T cell activation [36].

In work to identify a transcriptional signature to distinguish acute malaria from other febrile illnesses, Griffiths et al. compared the GEP of twenty-two Kenyan children admitted with febrile illnesses (fifteen of which had malaria infection alone) with six convalescent samples collected 2 weeks post discharge [23]. Two main GEPs relating to neutrophil and erythroid activity were shown to differentiate acutely ill and convalescent children, with significantly higher expression of genes in the neutrophil-related gene region in subjects with bacterial infections and significantly higher expression of genes related to lymphocyte and T cell activation in subjects with malaria. The authors also identified two gene profiles whose expression intensity correlated with host parasitaemia.

Only two datasets included gene expression changes following *P. vivax* infection [28, 30, 37]. Rojas-Penas et al. interrogated GEP changes in malaria naïve (MN) and malaria-exposed (ME) Columbian volunteers following infection with *P. vivax* in a CHMI setting [28]. Significant GEP changes were consistent with time-point rather than prior malaria exposure, with a decline in innate immune signalling and neutrophil number (in contrast to strong up regulation of the same genes reported by Igadour et al. [36]) and an increase in interferon induction seen at diagnosis. No significant GEP changes were noted at other time points, including those relating to the liver stage of infection. Further analysis of this dataset by Vallejo et al. using network co-expression analysis showed that while *P. vivax* infection induced strong inflammatory responses in all participants, the inflammatory response was attenuated with pathways associated with antigen processing and presentation less enriched in those with prior exposure to *P. vivax*, suggesting a more ‘tolerogenic’ immune response in these individuals [30].

In contrast to this work, Rothen et al. found that transcriptional changes post-CHMI via intradermal injection of cryopreserved *P. falciparum* sporozoites were most pronounced on day 5 after inoculation, during the clinically silent liver stage rather than during the blood-stage of infection [38].

### Transcriptomic studies in specific cell types

Whilst the majority of studies examined the immune response from whole blood or PBMCs, some examined transcriptomic changes in other cell types or tissues [26, 33, 39–45]. For example, the work of Muehlenbachs et al. with placental tissue highlighted a previously unappreciated role for B cells in chronic placental malaria [39]; whilst Sullivan et al. compared GEPs of classical and ‘atypical’ memory B cells obtained from Ugandan children showing the latter demonstrated down-regulation of B cell receptor signalling and apoptosis [43].

## Discussion

GEP is a powerful tool to analyse the immune response to infection. As this review demonstrates, the application of these studies for malaria are wide-ranging, from attempts to dissect the mechanisms of NAI to improving understanding of the interaction between host genotype and infection outcome. However, as a field in its relative infancy, studies are often hypothesis generating with extremely small sample sizes. There is a lack of standardization ranging from methodological (such as sample type, RNA extraction, platform and analysis) to phenotype (including precision in disease context and immune status). This variation means interpreting published data and comparison between studies is challenging. Some of this is unavoidable, however, much could be addressed, for example by implementing standardization in blood sampling, methodological protocols for data generation and analysis with robust significance testing and approaches to confounders, use of ontologies (for example human phenotype and gene ontologies) and expert curation and annotation of data on deposition [46–49].

GEP studies are well placed to examine the mechanisms of NAI and have already helped highlight the role of the innate and early adaptive immune responses [24–26]. However, work has been limited by the lack of an in vitro correlate or universally accepted definition of NAI, meaning identifying the immune status of individuals or quantification of immunity is problematic [6, 50]. In field studies where the timing of infection and parasite burden and dynamics are unknown, and potentially hugely variable between individuals, only limited information can be reliably extrapolated from any GEP changes seen. Most studies assess gene expression from peripheral blood or its components, which does not provide reliable information regarding the transcriptional changes in key organs such as spleen, liver, and bone marrow. In addition, when subjects are recruited at presentation with disease, no baseline comparator data are available to use as a control. Even if a clear difference in GEP were to be reported between individuals with and without NAI, it would be near impossible to distinguish GEP changes associated with parasitaemia from those mediating immunity.

However, there is much potential for the future use of GEP studies, particularly in CHMI studies [51, 52] where the parasite burden can be pre-defined and dynamics of infection closely monitored using highly sensitive qPCR. As these studies are increasingly performed in endemic settings [53–55], there will be growing opportunity to use GEP to understand detailed time-course changes in immune response, particularly at the skin, liver and pre-symptomatic blood-stage, which to date have been difficult to study in human subjects infected in the field.

## Conclusion

GEP in malaria is a potentially powerful tool, but to date studies have been hypothesis generating with small sample sizes and widely varying methodology. As CHMI studies are increasingly performed in endemic settings, there will be growing opportunity to use GEP to understand detailed time-course changes in host response and understand in greater detail the mechanisms of NAI.

## Supplementary information

**Additional file 1: Figure S1.** Flowchart summarizing identification of GEO datasets and publications.

## Data Availability

Data sharing is not applicable to this article as no datasets were generated or analysed during the current study

## Abbreviations

CHMI: controlled human malaria infection
GEO: gene expression omnibus
GEP: gene expression profile
NAI: naturally acquired immunity
PBMC: peripheral blood mononuclear cells
