# Supplementary material for: Use of gene expression studies to investigate the human immunological response to malaria infection
Source: Malar J. 2019 Dec 13;18:418. doi: 10.1186/s12936-019-3035-0 (PMC6911278; doi:10.1186/s12936-019-3035-0)
Supplement: Supplementary file 1 — Additional file 1: Figure S1. Flowchart summarizing identification of GEO datasets and publications. [file 12936_2019_3035_MOESM1_ESM.pdf]

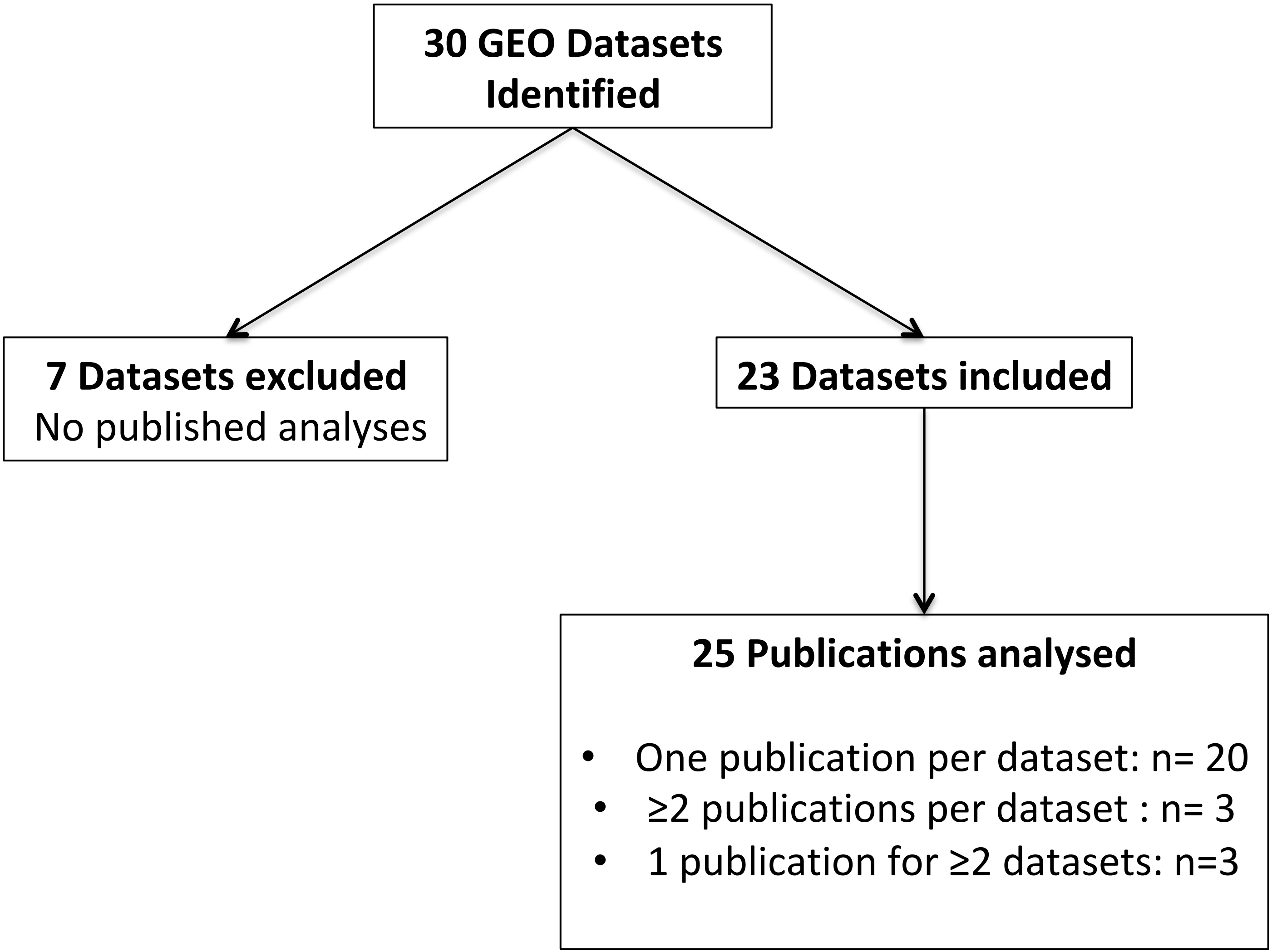
30 GEO Datasets Identified
7 Datasets excluded
No published analyses
23 Datasets included
25 Publications analysed
One publication per dataset: n= 20
≥2 publications per dataset : n= 3
1 publication for ≥2 datasets: n=3
